# Osteoclasts’ Ability to Generate Trenches Rather Than Pits Depends on High Levels of Active Cathepsin K and Efficient Clearance of Resorption Products

**DOI:** 10.3390/ijms21165924

**Published:** 2020-08-18

**Authors:** Xenia G. Borggaard, Dinisha C. Pirapaharan, Jean-Marie Delaissé, Kent Søe

**Affiliations:** 1 Department of Clinical Cell Biology, Vejle Hospital/Lillebaelt Hospital, 7100 Vejle, Denmark; dinisha2@hotmail.com (D.C.P.); Jean-Marie.Delaisse@rsyd.dk (J.-M.D.); 2 Department of Regional Health Research, University of Southern Denmark, 7100 Vejle, Denmark; 3 Clinical Cell Biology, Department of Pathology, Odense University Hospital, 5000 Odense C, Denmark; 4 Clinical Cell Biology, Pathology Research Unit, Department of Clinical Research, University of Southern Denmark, 5000 Odense C, Denmark; 5 Clinical Cell Biology, Department of Molecular Medicine, University of Southern Denmark, 5000 Odense C, Denmark

**Keywords:** cathepsin K, osteoclast, bone resorption, pit, trench, lysosome, transcytosis, clathrin, chloroquine, functional secretory domain

## Abstract

Until recently, it was well-accepted that osteoclasts resorb bone according to the resorption cycle model. This model is based on the assumption that osteoclasts are immobile during bone erosion, allowing the actin ring to be firmly attached and thereby provide an effective seal encircling the resorptive compartment. However, through time-lapse, it was recently documented that osteoclasts making elongated resorption cavities and trenches move across the bone surface while efficiently resorbing bone. However, it was also shown that osteoclasts making rounded cavities and pits indeed resorb bone while they are immobile. Only little is known about what distinguishes these two different resorption modes. This is of both basic and clinical interest because these resorption modes are differently sensitive to drugs and are affected by the gender as well as age of the donor. In the present manuscript we show that: 1. levels of active cathepsin K determine the switch from pit to trench mode; 2. pit and trench mode depend on clathrin-mediated endocytosis; and 3. a mechanism integrating release of resorption products and membrane/integrin recycling is required for prolongation of trench mode. Our study therefore contributes to an improved understanding of the molecular and cellular determinants for the two osteoclastic bone resorption modes.

## 1. Introduction

Osteoclasts (OCs) are multinucleated cells that form through fusion of mono-nucleated precursors of the myeloid lineage [1]. These are the only cells of the human body that can efficiently dissolve the mineral component through secretion of protons onto the bone surface. Demineralization exposes the vast network of large, complex, and inter-crosslinked collagen type I fibers that are present throughout all bones [2]. These complex fibers are only efficiently degraded by cathepsin K [3,4,5,6]. A prerequisite for these two essential processes to occur is that OCs form a so-called actin ring, generating a tight contact to the bone surface mediated by densely packed podosomes [7,8]. Within this actin ring, a sealed compartment is formed and a specialized membrane area, called the ruffled border, is generated. This ruffled border mediates both the secretion of protons and collagenolytic enzymes such as cathepsin K onto the bone surface, facilitating bone resorption. This process is dependent on a complicated transport system of vesicles such as secretory lysosomes. Secretory lysosomes are believed to supply the ruffled border with membranes containing, e.g., vacuolar-type H^+^-ATPase (v-ATPase) and chloride channel 7 (CLC7), but also collagenolytic enzymes such as a series of cathepsins, (in particular cathepsin K) that are released within the sealing zone. The dissolved mineral component and resorption products are taken up through endocytosis and the resulting vesicles are transported to the functional secretory domain (FSD) where the vesicles fuse with the cell membrane and release the contents [9].

Up until recently, it was widely accepted that OCs have to be immobile during bone resorption, but that they migrate between periods of resorption. This dogma is commonly recognized as the “bone resorption cycle” [10,11,12,13,14]. However, recently we demonstrated by time-lapse that human OCs can resorb bone in two different modes: 1. the intermittent and stationary resorption mode, resulting in rounded resorption cavities termed pits. This mode corresponds to the dogma of the “bone resorption cycle”. 2. The continuous resorption mode where the OC moves while it resorbs bone, at the same time resulting in deep elongated cavities termed trenches [15]. Not only are these two resorption modes very different with respect to movement, but they are also different with respect to resorption speed, resorption depth, and sensitivity to drugs [15,16,17,18,19,20]. Furthermore, it was recently shown that OCs generated in vitro from peripheral blood monocytes of women gained the capacity to make more trenches with increasing age of these women [21]. We only have little knowledge about what are the molecular and cellular determinants that make these two different resorption modes so different. We therefore still have many unanswered questions regarding the fundamental differences between these two resorption modes. Seen in this light, we set out to further elaborate on the differences between these two resorption modes.

We isolated human CD14^+^ monocytes, differentiated them into OCs in vitro, reseeded them onto bovine cortical bone slices, and used various inhibitors (odanacatib, chloroquine, and Pitstop2) to investigate potential differences between pit- and trench-resorption modes. This allowed us to address differences between these resorption modes with respect to extra- and intra-cellular degradation of collagen as well as its release. We show that OCs making trenches have a higher level of active cathepsin K and require a mechanism integrating the release of internalized resorption products and the detachment of the rear of the OC.

## 2. Results

### 2.1. OCs in Pit or Trench Mode Are Differently Sensitive to Drugs Affecting Collagenolysis, Acidification, and Endocytosis

**Figure 1:**
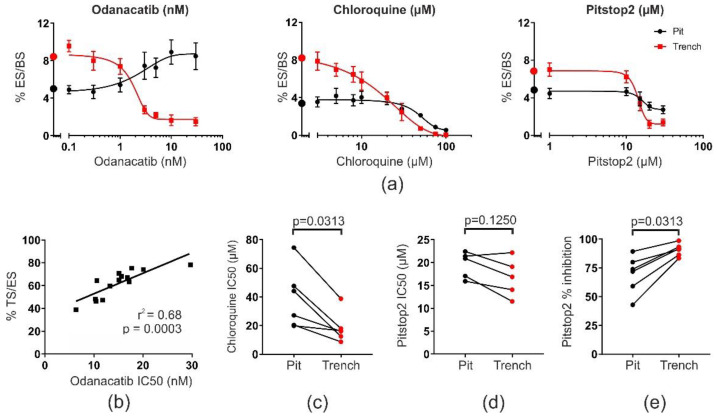
Different susceptibilities of pit- and trench-resorbing OCs to distinct types of inhibitors. (**a**) Representative dose–response curves of bone resorption upon treatment with three different inhibitors. Bone resorption is categorized into pit (black) and trench (red) surface per bone surface. Baseline levels are indicated by black and red circles on the y-axis, respectively. Data represent the results from a single experiment using OCs from a single donor (but different for each drug) and with *n* = 5 bone slices for each condition tested. %ES/BS, percent eroded surface per bone surface. Error bars represent SD. (**b**) Linear correlation between the mean trench surface per eroded surface (% TS/ES) (calculated from *n* = 5 bone slices) in control conditions and the respective IC50 (determined by curve fits as shown for odanacatib in (a)) of odanacatib determined in OC cultures from 14 different donors (each experiment was performed as shown in (a)). (**c**) Comparison of IC50-values obtained from six different experiments (using different donors) determined for pit surface per bone surface (black) and trench surface per bone surface (red). Data obtained from the same experiment are connected with a line. Each IC50 was determined from individual experiments based on curve fits as shown for chloroquine in (a). Statistics: Wilcoxon paired test. (**d**) Comparison of pit and trench IC50 values obtained from five different experiments (using different donors) after treatment with Pitstop2. Data obtained from the same experiment are connected with a line. Each IC50 was determined from individual experiments based on curve fits as shown for Pitstop2 in (a). Statistics: Wilcoxon paired test, *n* = 5. (**e**) Comparison of percentwise inhibition at the highest dose of Pitstop2 with respect to pit and trench surfaces obtained from six different experiments (using different donors). Data obtained from the same experiment are connected with a line. Each level of inhibition was determined from individual experiments based on curve fits as shown for Pitstop2 in (a). Statistics: Wilcoxon paired test.

We have previously published a study that confirms that OCs making trenches or pits resorb bone at very different speeds [15,16,17,22]. In order to better understand what the reason for these differences may be, we used three different inhibitors affecting the essential steps during osteoclastic bone resorption (Figure 1a): 1. Odanacatib, a specific active-site inhibitor of cathepsin K, the main enzyme responsible for collagenolysis. Odanacatib is designed to specifically act on cathepsin K in the extracellular resorption compartment [23]. 2. Chloroquine, a lysosomotropic agent, which accumulates and initially raises the pH in intracellular acidic compartments despite active v-ATPase activity [24,25,26]. These acidic intracellular compartments are necessary for clearance of resorption products [9]. 3. Pitstop2, an inhibitor of clathrin-mediated endocytosis [27].

Figure 1a (left) shows a titration with odanacatib using OCs generated from one donor. It is clearly seen that this drug primarily inhibits OCs making trenches, while those making pits are concomitantly increased, just as previously observed [18]. The consequence is that the total eroded bone surface only changes marginally, but that the proportion of eroded surfaces covered by trenches is dramatically affected. This suggests that cathepsin K is most active in OCs making trenches. Due to the binding of odanacatib to the active site of cathepsin K, the half maximal inhibitory concentration (IC50) indirectly reflects the number of active cathepsin K enzymes in e.g., OCs making trenches. We have previously observed that the expression of cathepsin K varies when analyzing OCs generated from different donors and also the proportion of trench surface per eroded surface [22]. Therefore, we performed IC50 determinations (as shown in Figure 1a, left) using blood from 14 different blood donors (Figure 1b). We found that there is a strong linear correlation between the number of active cathepsin K enzymes in a given cell culture and the extent of eroded surfaces made up by trenches (%TS/ES) (Figure 1b), irrespective of the total eroded surface. The percent trench surface per eroded surface varied from 46 to 78%, and 68% of this variation can be explained by differences in the levels of active cathepsin K (Figure 1b).

In contrast to odanacatib, chloroquine inhibits both OCs making trenches as well as those making pits. However, the titration with chloroquine shown in Figure 1a (center) suggests that OCs making trenches already are sensitive to chloroquine at concentrations below 20 µM, whereas those making pits are mostly not. This leads to a lower IC50 for trench-making OCs than those making pits. Figure 1c shows that this observation can be repeated when using blood from six different blood donors since the IC50 of OCs making trenches is systematically lower than those making pits. Pitstop2 (selective inhibitor of clathrin-mediated endocytosis) inhibited both OCs in pit and trench mode with similar IC50s (Figure 1a, right), which was also confirmed in five independent experiments (Figure 1d). However, Pitstop2 was able to more effectively inhibit the absolute resorption levels of OCs making trenches than those making pits (Figure 1a, right, and Figure 1e).

### 2.2. Chloroquine (at 10 or 20 µM) Does Not Affect Resorption Depth but Shortens Trenches Significantly

**Figure 2:**
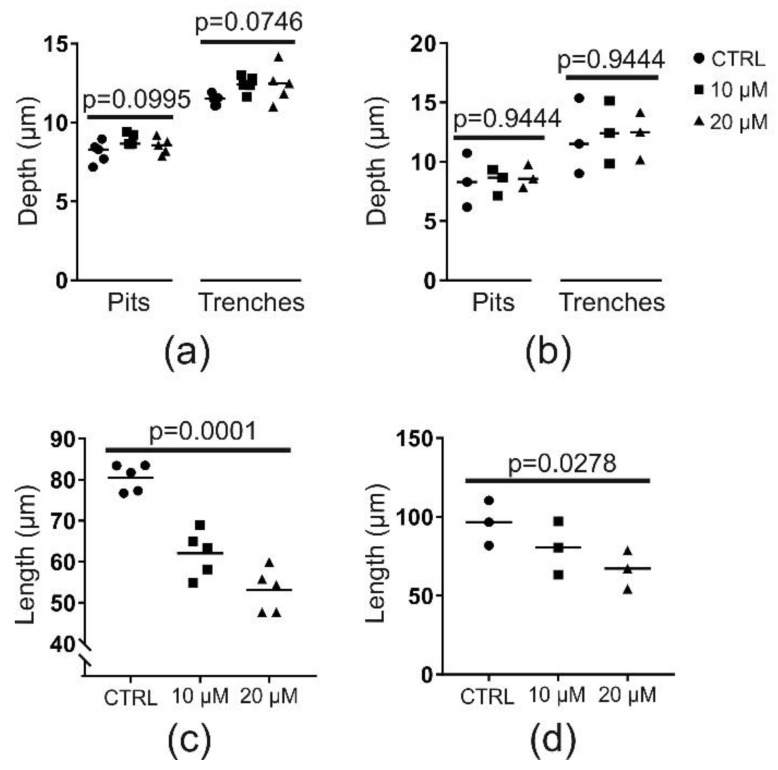
Chloroquine, at 10 or 20 µM, does not reduce the demineralization depth of pits or trenches, but significantly reduces the length of trenches. (**a**) Representative analysis of depth from one resorption experiment (using OCs from a single donor) subdivided into pits and trenches. Each point represents the mean of 100 analyzed events per bone slice (*n* = 5 bone slices per condition). Statistics: Kruskal–Wallis test. (**b**) Median depth of pits and trenches in three different experiments with OCs from different donors. These medians were determined from five bone slices per condition as shown in (a). Statistics: Friedmann test. (**c**) Representative analysis of trench length from one resorption experiment (using OCs from a single donor); each point represents the mean of 100 events per bone slice (*n* = 5 bone slices per condition). Statistics: Kruskal–Wallis test. (**d**) Median length of trenches in three different experiments with OCs from different donors. These medians were determined from five bone slices per condition as shown in (c). Statistics: Friedmann test.

Since we aim at identifying which mechanistic characteristics are specific to trench mode, we focused on the effects of chloroquine at concentrations below 20 µM, which primarily affect trench mode. First, we asked whether the target of chloroquine at these concentrations was intra- or extracellular. To do so, we determined the average demineralization depths. Our rationale was that if chloroquine would affect the pH in the sub-osteoclastic resorption compartment, it would compromise the maximum demineralization depths. Chloroquine did not compromise demineralization in an experiment using OCs generated from a single blood donor (Figure 2a). This was confirmed when comparing the outcome of three independent experiments (Figure 2b). This suggests that chloroquine targets the OC intracellularly rather than the resorptive space. However, chloroquine caused a significant and dose-dependent decrease in the length of trenches (Figure 2c,d). The latter finding implies that chloroquine may especially inhibit OCs making trenches by affecting resorption speed and/or duration of resorption. In order to thoroughly investigate these possibilities, we analyzed bone-resorbing OCs by time-lapse recording in the absence or presence of chloroquine.

### 2.3. Chloroquine (10 µM) Does Not Affect Resorption Speed but Induces a Premature Stop of Resorption by OCs in Trench Mode

**Figure 3:**
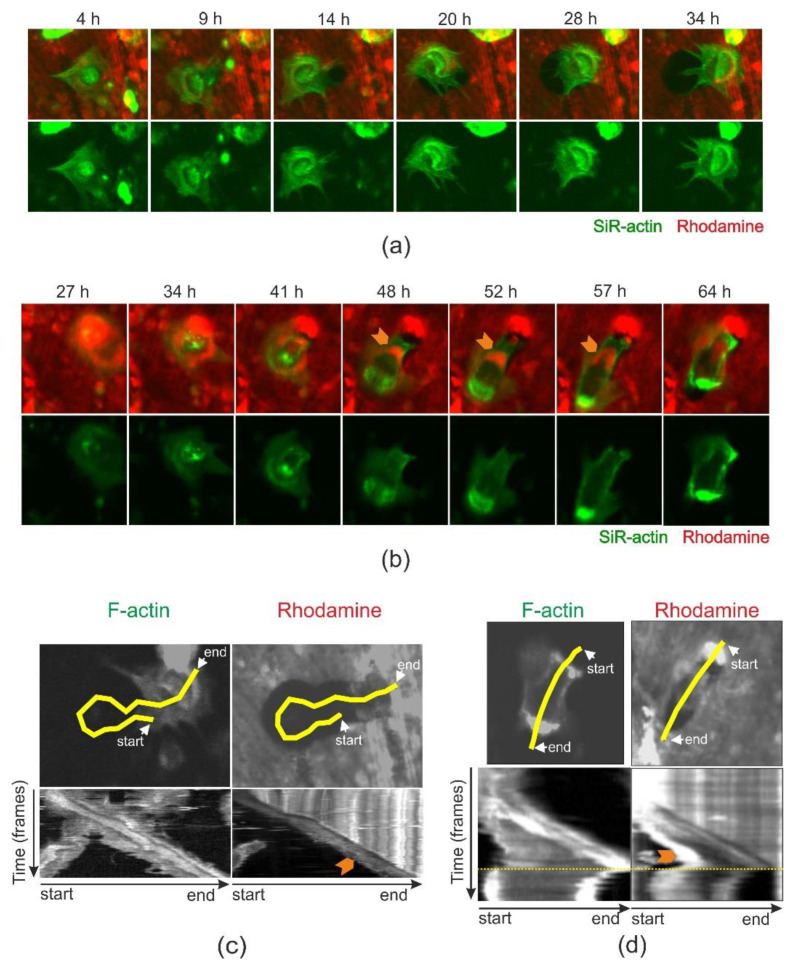
Time-lapse analyses document that 10 µM chloroquine makes trench-forming OCs stop prematurely. Representative images selected from one 70 h time-lapse recording. (**a**) Images from control condition (Supplementary Materials Movie 1) showing selected time-lapse images out of 70 h total recording time. The images show an OC making a pit and subsequently switching into trench formation. The width of each image is 122.6 µm. (**b**) Images from the chloroquine condition (10 µM chloroquine added 4 h prior to start of recording, Supplementary Materials Movie 2), showing selected time-lapse images from a total recording of 70 h. The images show an OC making a pit and subsequently switching into trench formation. The width of each image is 176.4 µm. (**c**) Kymograph analysis of the control OC shown in (a) and Supplementary Materials Movie 1 showing SiR-actin or rhodamine signals throughout the time-lapse recording. The upper images show the trace line (yellow) that reflects movement of the OC (left) or the resorption front (right) over time and along which the fluorescent intensities are measured. The images reflect the end stage of the time-lapse recording. The lower images show the kymographs reflecting the intensity of f-actin or rhodamine over time along the yellow line. The absence of accumulated rhodamine signal in the OC is marked by an orange arrow. (**d**) Kymograph analysis of the chloroquine treated OC shown in (b) and Supplementary Materials Movie 2 showing SiR-actin or rhodamine signals throughout the time-lapse recording. The upper images show the trace line (yellow) that reflects movement of the OC (left) or the resorption front (right) over time and along which the fluorescent intensities are measured. The images reflect the end stage of the time-lapse recording. The lower images show the kymographs reflecting the intensity of f-actin or rhodamine over time along the yellow line. The time point where the front of the resorbing OC detaches and resorption stops is marked by a narrow yellow dotted line. The accumulation of rhodamine signal in the OC just prior to termination of bone resorption is marked by an orange arrow.

When analyzing time-lapse recordings, it is evident that OCs making trenches under control conditions continued resorbing once started (Figure 3a, Supplementary Materials Movie 1), in line with earlier observations [15]. In contrast, however, OCs exposed to chloroquine suddenly terminated bone resorption (Figure 3b, Supplementary Materials Movie 2). This is further highlighted by comparing the kymographs for the OC shown in Figure 3a and Supplementary Materials Movie 1 (Figure 3c) and Figure 3b and Supplementary Materials Movie 2 (Figure 3d). When analyzing the actin and rhodamine signals in more detail for control condition, it is seen that the front actin signal (reflecting the front sealing zone and the ruffled border) and the removal of rhodamine (also seen as a flow of intracellular vesicles trafficking towards the rear) progress at a constant rate, whereas the rear actin signal progresses irregularly. This fits exactly earlier observations [15]. Interestingly, chloroquine completely prevents the progression of the rear signal, but, initially, not that of the front signals that move ahead like in the control. This makes the OC stretch until it suddenly retracts making the front actin signal move backwards (reflecting detachment of the leading edge). The rear actin signal then intensifies. Even more interestingly, chloroquine does not prevent removal of rhodamine from the bone surface as long as the front of the OC (signal) moves ahead, but rhodamine gradually accumulates at the rear of the OC (Figure 3b,d), in contrast to control conditions where no accumulation is seen (orange arrowhead in Figure 3a,c).

**Figure 4:**
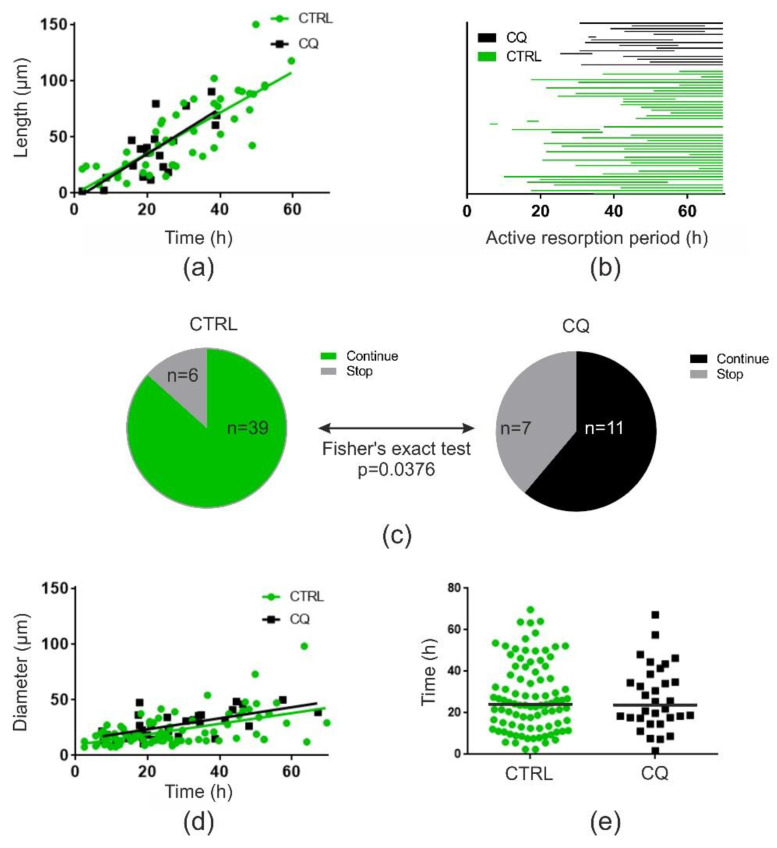
Quantification of time-lapse recording: Chloroquine (10 µM) reduces the duration of resorption but not the speed of OCs making trenches; OCs making pits are unaffected, illustrated by data obtained from time-lapse recordings with OCs from a single donor. (**a**) Relation between length of trench and time of active resorption for all trenches identified in one experiment, subdivided into control (CTRL; green; *n* = 47) with a linear regression fit of *r*^2^ = 0.65 and *p* < 0.0001 and chloroquine-treated (CQ; black; *n* = 20) with a linear regression fit of *r*^2^ = 0.57 and *p* = 0.0001. Slopes and y-intercepts of both linear regression fits were tested and found not to be different (*p* = 0.6738 and *p* = 0.9678, respectively) (**b**) Diagram visualizing the timeframes of active resorption for individual OCs making trenches, subdivided into control (green; *n* = 45) and chloroquine-treated (black; *n* = 18). (**c**) Pie chart showing the percentage of trench-making OCs that stop resorption during time-lapse recording (grey) compared to those that continue throughout recording in control (green) and chloroquine (grey) conditions. Statistics: Fishers exact test (*p* = 0.0376). (**d**) Relation between diameter of pits and time of active resorption for all pits identified in one experiment, subdivided into control (green; *n* = 86) with a linear regression fit of *r*^2^ = 0.28 and *p* < 0.0001 and chloroquine-treated (black: *n* = 31) with a linear regression fit of r^2^ = 0.40 and *p* < 0.0001. Slopes and y-intercepts of both linear regression fits were tested and found not to be different (*p* = 0.9075 and *p* = 0.0622, respectively). (**e**) Active resorption time for all pits identified in one experiment for control (green; *n* = 91) and chloroquine (black; *n* = 32). Statistics: Mann–Whitney test (*p* = 0.9999).

Data shown in Figure 3 represent isolated observations, but when analyzing all events (a total of 270 events) from the same experiment, we could confirm that the resorption speed was unaffected both for OCs making trenches (Figure 4a) and pits (Figure 4d). OCs making trenches stopped prematurely when exposed to 10 µM chloroquine (Figure 4b,c), whereas this was clearly not the case for OCs making pits (Figure 4d,e). In fact, OCs making trenches under chloroquine exposure were 4-fold more likely to stop before the end of recordings (odds ratio: 4.1, Fischer’s exact test) than those under control conditions (Figure 4c).

### 2.4. Cathepsin D-Containing Lysosomes Appear at the Resorptive Front of Trench-Forming OCs and Are Transported Towards the Distal End

**Figure 5:**
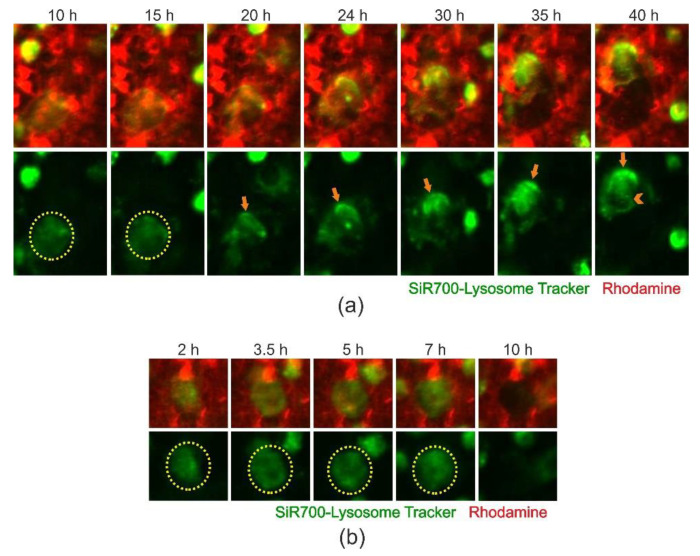
Time-lapse recording reveal that cathepsin D positive lysosomes show different abundance and transportation in OCs making trenches and pits. Images are selected snapshots from Supplementary Materials Movie 3 showing a 70 h time-lapse recording of resorbing OCs labelled with SiR700-Lysosome Tracker on rhodamine coated bovine bone slices. (**a**) An OC initially making a pit (yellow circle) and subsequently switching into trench mode resorption. Orange arrows indicate the resorption front while the orange arrow-head highlights where the lysosomes are transported to at the rear end of the OC. The width of each image is 72.4 µm. (**b**) An OC only making a pit. Cathepsin D-positive vesicles are distributed equally within the boundaries of the cavity (yellow circle) without any signs of polarization. The width of each image is 56.6 µm.

Time-lapse recordings of bone-resorbing OCs labelled with a probe binding cathepsin D, a lysosomal marker, revealed a strong and pronounced polarization of cathepsin D-containing vesicles at the resorptive front (Figure 5a, orange arrow; Supplementary Materials Movie 3). Movie 3 (Supplementary Materials) also clearly shows transportation of cathepsin D-containing vesicles from the resorption front towards the rear end of the trench-forming OC, where the tracing/labeling was lost (Figure 5a, orange arrow-head). In pit-forming OCs, no polarization of cathepsin D-containing vesicles was observed as resorption progressed (Figure 5b, Supplementary Materials Movie 4). Thus, it is clear that lysosomes are very prominent in OCs making trenches during resorption and are transported to the rear end of the resorbing OC.

### 2.5. Chloroquine Accumulates in the Distal End of Trench-Forming OCs

**Figure 6:**
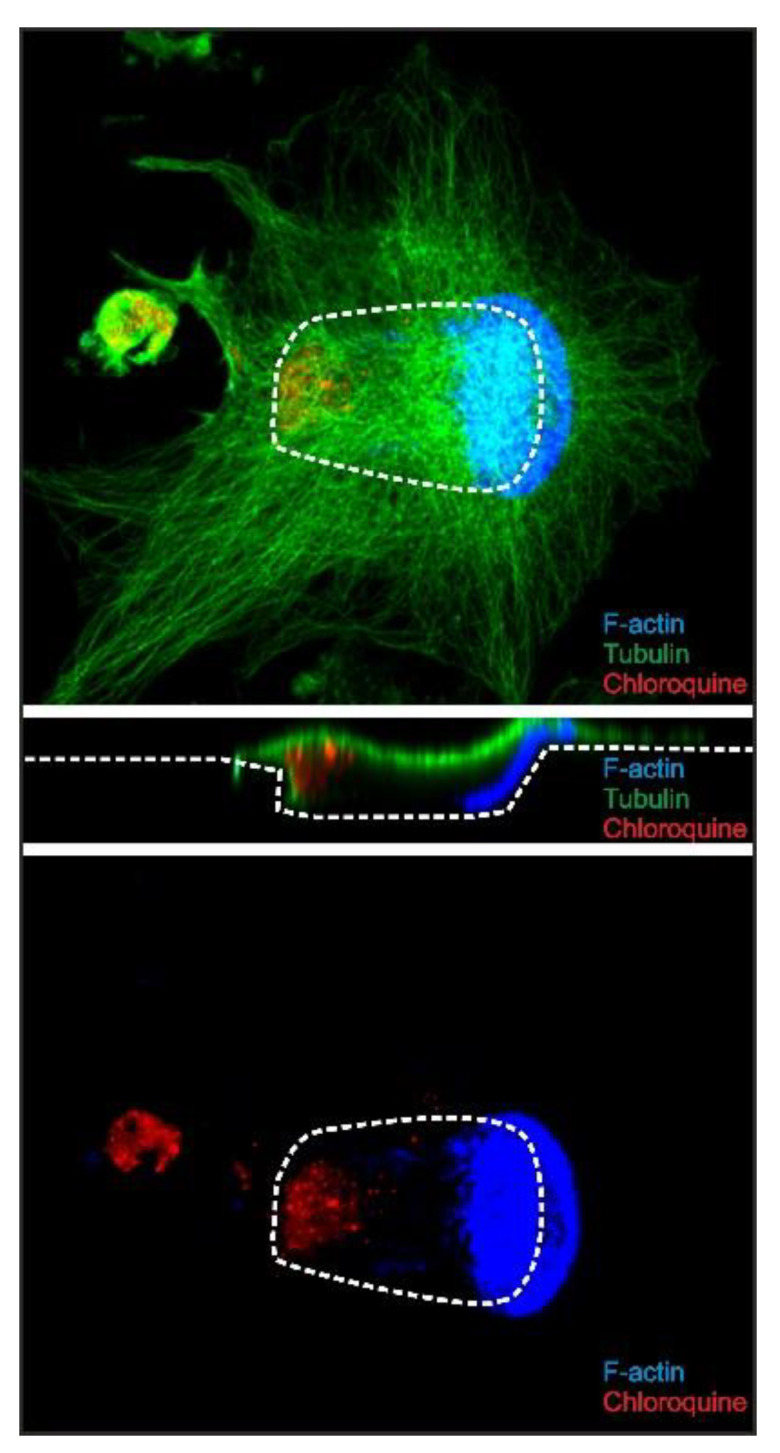
Chloroquine accumulates towards the rear end of trench-making OCs. Chloroquine immunoreactivity is shown in red, f-actin in blue, and tubulin in green. Upper and lower images are views looking down on the OC from above the bone surface, whereas the center image shows a longitudinal cross-section of the same OC. Dashed white lines show the edge of the resorption cavity (top and bottom images), while it shows the bone surface in the center image. The width of all three images is 141.4 µm.

**Figure 7:**
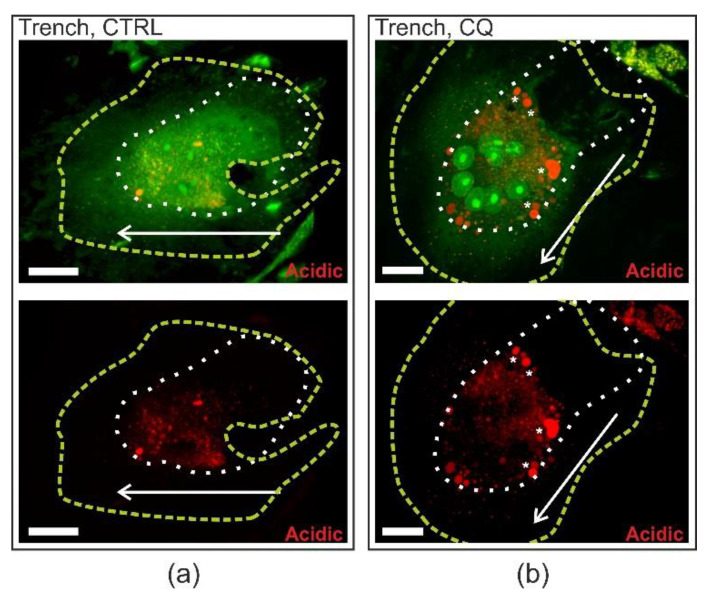
Chloroquine seems to cause an enlargement of acridine orange stained vesicles in OCs making trenches. (**a**) A 3D confocal view of trench-forming OC in the control condition. (**b**) A 3D confocal view of a trench-forming OC treated with 10 µM chloroquine. Circumference of OCs are indicated by a green dashed line, while the edge of the resorptive cavities is shown with a white dashed line. The direction of resorption is indicated with an arrow. Acridine orange turns red/orange in acidic compartments, while in more neutral compartments it is green. The white bars represent 20 µm.

Figure 2 shows that resorption depths of trenches were unaffected, and Figure 3 and Figure 4 demonstrate that resorption speed was unaffected. These observations and the fact that chloroquine is lysosomotropic may suggest that chloroquine will not mediate its effect at the resorption front. Combining immunofluorescent staining of chloroquine with confocal imaging of resorbing OCs treated with 10 µM chloroquine, we found that chloroquine-containing vesicles accumulated at the distal end of trench-forming OCs (Figure 6). The profile view highlights that chloroquine accumulates inside the cell especially at the rear end and not in the extra cellular resorption zone or at the resorption front (Figure 6). We could not detect this chloroquine distribution pattern in pit-forming OCs (not shown). Regarding the pattern of chloroquine in trench-forming OCs, it is interesting that acridine orange staining of live, unfixed, and bone-resorbing OCs suggests that acidic vesicles may become larger in chloroquine-exposed trench-making OCs compared to untreated ones (Figure 7).

## 3. Discussion

For decades, it has been commonly accepted that bone resorption by OCs is always executed through one mechanism where the OC would be immobile during bone resorption, making pits. However, recently a series of publications have documented that there is not just one way for OCs to resorb bone, but two. It has for long been known that two different shapes of resorption cavities exist in vivo, namely pits (rounded resorption cavities) and trenches (longitudinally extended resorption lacunae) [22,28,29,30,31]. Although these very different shapes of resorption cavities have been recognized for decades, they were over the years thought to be made through the same type of resorption process—explained through the “resorption cycle” model—where osteoclastic bone resorption would go through alternating stages of bone resorption, migration, and reinitiating resorption at a new site [10,11,12,13,14,32]. It was not until recently that time-lapse recording of actively resorbing human OCs in vitro demonstrated that these differently shaped cavities represent two resorption modes of OCs—trench mode and pit mode—differing in resorption speed, depth, and duration, thus clearly showing a difference in aggressiveness [15]. These differences obviously reflect differences in the resorption machinery, some of which are identified herein.

### 3.1. High Levels of Cathepsin K Are Needed for Initiating Trench Formation: Th Role of Extracellular Collagenolysis

Our earlier study showed that 90% of the trenches start as a pit [15] and that sufficient cathepsin K activity is an absolute prerequisite to switch from pit to trench mode. The main evidence supporting this finding is that increasing concentrations of odanacatib in bone resorption assays lead to decreasing trench surface (down to an almost complete abolishment at higher concentrations) and concomitantly increasing pit surface [18,33]. This concomitant effect on trench and pit formation is confirmed here. Interestingly, these dose–response curves provide a way to titrate active cathepsin K in resorbing OCs in vitro generated from different donors. These titrations allowed us here to further demonstrate a strict correlation between the levels of active cathepsin K (evaluated through IC50) and the prevalence of trench formation in a cohort of 14 donors. The levels of active cathepsin K actually explain roughly 70% of the variation in trenches per eroded surface. Thus, the cathepsin K level is a key determinant for the aggressiveness of OCs generated in vitro from different individuals. This is important to know because cathepsin K expression levels strongly vary in OCs generated in vitro from different individuals compared to other OC proteinases and depend on gender [22]. Furthermore, the levels of active cathepsin K in human OCs in vitro were recently reported to increase with the age and menopause status of the donor [21].

Mechanistically, the key contribution of cathepsin K to bone resorption is collagen degradation [3]. Accordingly, cathepsin K-rich OCs making trenches leave very little undegraded demineralized collagenous matrix at the bottom of the trenches, whereas cathepsin K-poor OCs making pits leave variable, but significant amounts of undegraded demineralized collagenous matrix at the bottom of the pit [17,33,34]. Because collagen is a negative regulator of OC bone resorptive activity [35,36,37], these observations led to a model where accumulation of demineralized collagen makes the OC stop resorbing and migrating right away, so that cathepsin K-poor OCs perform intermittent resorption (alternations of resorption and migration) [16,17]. In contrast, absence of accumulation of undegraded collagenous matrix lets the OCs resorb continuously, so that cathepsin K-rich OCs resorb over prolonged periods thereby generating long trenches [16,17]. These observations emphasize that the specific pathway linking cathepsin K levels and the decision of making a trench or a pit, is the lysis of the collagenous matrix in the sub-osteoclastic resorption compartment by cathepsin K. However, since our evaluations indicate that the levels of active cathepsin K explain roughly 70% of the overall prevalence of trench surface, other factors are also expected to influence this prevalence.

### 3.2. Involvement of Clathrin-Mediated Endocytosis in Both Pit- and Trench-Forming OCs

The role of the ruffled border is not limited to bone degradation. It also clears away the resorption products [38,39]. The latter activity is known to involve clathrin-mediated endocytosis. It is believed to occur at the central zone of the ruffled border in pit-forming OCs and at the rear zone of the ruffled border in trench-forming OCs, based on the abundance of clathrin immunoreactivity at these respective positions [40]. These same zones showed immunoreactivity of dynamin [40], which is in line with the fact that dynamin pinches off the clathrin-coated cavitations, thereby releasing intracellular vesicles [41]. In this context, it is interesting to note that dynamin-overexpressing OCs make long resorption trenches instead of round pits on dentine slices [42], suggesting that the uptake mechanism of resorption products may have distinct characteristics in trench mode compared to pit mode. Supporting this view, clathrin and dynamin immunoreactivity appeared stronger in trench-forming OCs than in pit-forming OCs [19,40]. Based on these observations, we hypothesized that an inhibitor of clathrin-mediated endocytosis would preferentially inhibit trench formation. Pitstop2 is reported to be such an endocytosis inhibitor, blocking ligand association with the N-terminal domain of clathrin [27], and to the best of our knowledge, Pitstop2 has never been tested on the resorptive activity of OCs. Our current study shows that Pitstop2 inhibits pit and trench formation with similar IC50s, but reaches a higher maximum inhibition of trench formation (higher than 80%). However, the interpretation of differences in maximum inhibition is not straight forward, as collagen relative to mineral removal is very variable in pits [17,18]. Therefore, the need for endocytosis is smaller if little collagen is degraded and therefore the response to Pitstop2 may be smaller. Thus, Pitstop2 allowed us to demonstrate a critical involvement of clathrin-mediated endocytosis in OCs, but it did not allow us to detect major differences between the trench and pit resorption modes at the level of uptake of resorption products by the ruffled border.

### 3.3. A Mechanism Integrating Release of Resorption Products and Membrane/Integrin Recycling Is Needed for Prolonged Simultaneous Resorption and Migration

A remarkable property of trench mode is that once OCs have started making a trench, they are unlikely to stop elongating it: only 14% and 13% of the trench-forming OCs stopped within the 72 h culture periods in our previous [15] and present study, respectively. It is therefore interesting that the presence of chloroquine increases the risk of discontinuation by four-fold. In addition, this effect is observed at 10 to 20 µM chloroquine concentrations, at which pit formation is only marginally affected. Thus, within this concentration range, the target of chloroquine is fairly specific for trench mode and thus contributes to decreasing the prevalence of trench surface relative to pit surface. Our data also indicate that the process targeted by 10 to 20 µM chloroquine relates quite specifically to prolongation of trench formation, since chloroquine does not appear to affect other resorption parameters such as resorption depth, the ability to start a trench, or the resorption rate whether in pit or trench resorption mode. How well can we define this specific target in trench-forming OCs? Chloroquine is well known to diffuse into acidic compartments of the cell, thereby raising their pH [43]. The most prominent acidic compartment in OCs is the extracellular space under the ruffled border. The current study clearly shows that this compartment is not targeted by chloroquine, since demineralization depths (conditioned by pH) are not affected. Furthermore, chloroquine does not have electronegative charges as required to diffuse into the sub-osteoclastic resorption compartment [44]. Instead, our immunostainings detect chloroquine in a population of intracellular vesicles that can be characterized as follows: 1. they appear to be rather large; 2. they are positioned at the rear of trench-forming OCs; and 3. this position coincides with the position where chloroquine induces the accumulation of resorption products as evaluated through rhodamine-labeled collagen in the time-lapse analyses and corresponding kymographs. Of note, in the absence of chloroquine, time-lapse does not detect an accumulation of resorption products, but instead shows a traffic of these products from the front to the rear of the OC where they disappear. This is in line with the concept that resorption products that are endocytosed into vesicles at the ruffled border cross the cytoplasm and are released at the opposed side [9,45,46]—which in pit-forming OCs corresponds to the top membrane and to the rear of a trench-forming OC. This specialized zone where this release occurs is the FSD [38,47,48]. Transcytosis to the FSD is a complex activity as the vesicles are modified and “mature” during their transportation [46]. This maturation is still poorly understood and includes fusion with other vesicles: e.g., tartrate-resistant acid phosphatase positive (TRAcP^+^) vesicles arising from the Golgi, and other vesicles, of which some are acidic and others not [49,50]. These fusions may lead to alterations of the cargo. However, there is no functional evidence showing which maturation characteristics of the vesicles are permissive for their release at the FSD—except that their dimension needs to be below a threshold size (Nathan J. Pavlos, University of Western Australia, Perth, Australia, personal communication). The molecular mechanism of the release itself is not known. There are thus many unknowns, rendering it difficult to interpret the specific effects of chloroquine.

The mode of action of chloroquine on cellular activities is also far from clear [41]. According to systematic studies conducted in a series of different cell types, the diffusion of chloroquine into acidic compartments goes along with a raise in pH, which is only transient and, importantly, the targeted compartments swell, probably through osmosis [43,51,52,53]. A series of functional alterations follows this swelling. This includes impaired fusion of lysosomes with autophagosomes, thereby blocking the delivery of sequestered cargo to lysosomes [52,53].

Yet, whatever the primary effect of chloroquine, our chloroquine experiments highlight that exocytosis of resorption products seems to be a necessity for prolongation of resorption. This could be interpreted as being caused by an intra-OC overload of resorption products [34], but also as a need for membrane recycling, since it has been emphasized that ruffled border membrane used for endocytosis of resorption products must be restituted, because maintaining the ruffled border function demands large amounts of membrane [54].

When searching for answers to why the effect of 10 to 20 µM chloroquine is specific for trench OCs, it is necessary to also consider that chloroquine selectivity could arise from an effect on migration rather than on resorption. Our previously published actin kymographs have stressed that the sealing zone and the ruffled border of trench-forming OCs move at a constant rate, whereas the rear is progressing irregularly [15]. This shows that the movement of the rear is governed in part independently from the movement of the front. The present blockade of movement of the rear of trench-forming OCs by 10 to 20 µM chloroquine further stresses the existence of a mechanism responsible for the coordination between the movement of the rear and the front. Similar observations were reported for OCs migrating over a surface without resorbing [55]: their leading edge moves ahead at a constant rate, whereas the rear is successively stretching and retracting. Retraction of the rear of these purely migrating OCs was shown to involve the disassembly of adhesion complexes with integrins [55], much in the same way as reported for migration of other cell types [56,57]. α_v_β_3_ integrins are present at high densities at the rear of OCs in trench mode [40] and at the top of the basolateral domain in pit mode [54] (i.e., the expected respective FSD site). α_v_β_3_ at the rear position is likely to uniquely influence the physical interactions with the bone surface compared with the pit resorption mode. In addition, it is well-known that integrins must recycle to the front to allow cell migration [57,58,59], otherwise the cell remains attached at the rear. Interestingly, in this respect, chloroquine not only causes attachment of the rear of the OC whose front stretches away, but also sudden detachment of this front, thus suggesting insufficient adhesion. Overall, the best interpretation of these observations is that integrin recycling is critical for the trench resorption mode.

**Figure 8:**
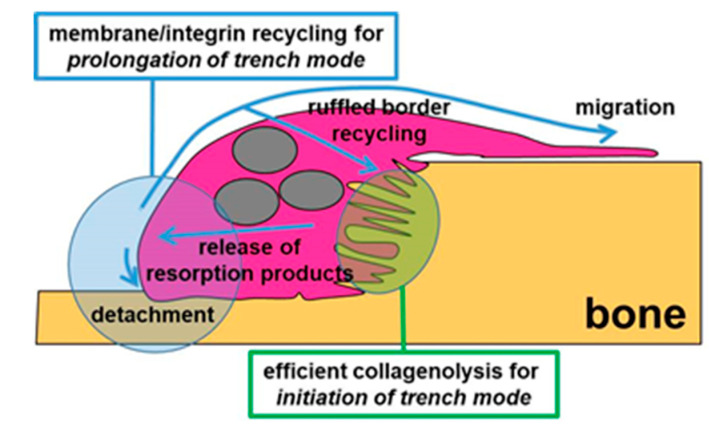
Model illustrating the unique features of the trench resorption mode demonstrated in the present study. Grey symbols represent three nuclei. Please refer to Discussion for further details.

Thus, we show herein that chloroquine concomitantly induces (i) arrest of OC migration as a result of the attachment of the rear and (ii) an arrest in the release of resorption products at the rear (concomitance shown very clearly in the kymographs). Taken together with the current knowledge on osteoclastic resorption and cell migration, this concomitance points to a model where the rear of the OC in trench mode functions as a sink for membrane/integrin recycling to the leading edge to enable migration, and for membrane recycling to the ruffled border, enabling bone resorption (Figure 8). A connection between clearance of resorption products and OC migration through membrane/integrin cycling is thereby revealed—which is likely to contribute to the unique efficiency of the trench resorption mode.

## 4. Materials and Methods

### 4.1. Generation of OCs

Blood was obtained from anonymous human male donors after obtaining an informed written consent (approved by the local ethical committee, S-20070019). Blood donors belong to the regular blood donor corps of Lillebælt Hospital and are in general between the ages of 18 and 67 years old according regulations by the Danish health authorities. Monocytes were isolated by gradient centrifugation through Ficoll-paque (GE Healthcare, Uppsala, Sweden). The CD14^+^ monocytes were isolated from cell-suspension after 30 min incubation at room temperature using BD IMag^TM^ Anti-Human CD14 magnetic particles (dilution 1:10), BD biosciences, San Jose, CA, USA) and subsequent washing on a magnetic device, according to the supplier’s instructions. Subsequently, the isolated cells were seeded in T75 culture flasks at a density of 5 × 10^6^ per T75 in culture medium (αMEM, ThermoFisher Scientific, Waltham, WA, USA) with 10% fetal bovine serum (FBS, Sigma-Aldrich, Darmstadt, Germany), 1% PenStrep (Sigma-Aldrich) and cultured at 37 °C and 5% CO_2_ in a humidified atmosphere. The CD14^+^ monocytes were differentiated into mature OCs using macrophage colony-stimulating factor (M-CSF) and receptor activator of nuclear factor kappa-B ligand (RANKL) according to our well established procedures [16,19,60]. In brief, CD14^+^ monocytes were exposed to 25 ng/mL M-CSF for two days followed by exposure to both 25 ng/mL M-CSF as well as 25 ng/mL RANKL for an additional 7 days (medium change twice), where after they were multinucleated (average number of nuclei per OC varied roughly between 4 and 8 nuclei) and considered to be “mature” OCs.

### 4.2. Osteoclastic Bone Resorption Assay

Matured OCs were harvested by accutase treatment (Biowest, Nuaillé, France) and resuspended in culture media with 25 ng/mL M-CSF and 25 ng/mL RANKL. The viability of detached cells was monitored with trypan blue staining prior to reseeding (in general viability varied between 75 and 90%). Resuspended OCs were seeded in 96-well plates on 0.4 mm thick bovine cortical bone slices (Boneslices.com, Jelling, Denmark) at a density of 1 × 10^5^ viable cells per bone slice. After 1 h of incubation at 37 °C and 5% CO_2_ in a humidified atmosphere, the inhibitor or corresponding vehicle solution was added to each well. At least four replicates were prepared per condition. Treated OCs were incubated for three days at 37 °C and 5% CO_2_ in a humidified atmosphere. After incubation, 150 µl conditioned media from each well was stored at −20 °C for TRAcP activity-analysis. Viabilty was determined by the use of CellTiter-Blue reagent (Promega, Madison, WI, USA) according to instructions by supplier. After CellTiter-Blue, OCs were lysed and bone slices were polished in ddH_2_O with a cotton swab, washed, dried and stained with toluidine blue (1% toluidine blue (*w*/*v*) in 1% sodium borate (*w*/*v*)).

### 4.3. Quantification of Bone Resorption

Toluidine blue-stained bone slices were analyzed by light microscopic examination and quantifications were performed in a blinded fashion using a random systematic count distinguishing between pits and trenches as described elsewhere [16,17,22]. Length of trenches was measured using the CellSens Entry software 1.14 (Olympus Life Science Solutions, Tokyo, Japan). Demineralization of the depths of the pit and trenches was performed as described elsewhere [16,17], performing a blinded analysis of 100 pits and 100 trenches per bone slice in a random systematic count.

### 4.4. IF Staining and Confocal Microscopy of Bone-Resorbing OCs

Matured OCs were seeded in 96-well plates on 0.2 mm thick cortical bovine bone (Boneslices.com, Jelling, Denmark) with a density of 1 × 10^5^ cells per bone slice in culture medium. After three days of incubation, OCs were washed in PBS and fixed in 3% (*w*/*v*) paraformaldehyde and 2% (*w/v*) sucrose for 15 min at room temperature. Fixed OCs were permeabilized and blocked for unspecific staining in PBS 0.5% (*w/v*) BSA and 0.05% (*w/v*) saponin and were subsequently incubated with primary antibody for 1 h and secondary antibody for 1 h, followed by co-staining with phalloidin Alexa Fluor 568 (ThermoFisher Scientific). The fixed and stained OCs were mounted using ProLong Gold with or without DAPI (ThermoFisher Scientific) and captured in z-stack mode using a FV10i confocal microscope (Olympus, Tokyo, Japan). The captured images were analyzed using Imaris 7.6 software (Bitplane, Zürich, Switzerland). Primary antibodies used: rabbit monoclonal anti-human tubulin (IgG, ab52866, Abcam Cambridge, UK) diluted 1:250 and mouse monoclonal anti-chloroquine antibody (IgG2a, ab23486, Abcam) diluted to a ratio of 1:500. To allow for a specific detection of chloroquine, each experiment contained a negative control (where OCs were not treated with chloroquine) was included. The level of background signal was used to subtract background from the OCs treated with chloroquine. Background was a weak diffuse signal, whereas specific signal appeared as foci. Secondary antibodies used: Alexa Fluor 647 goat anti-mouse (IgG2a, 115-605-206, Jackson Labs, West Grove, PA, USA) diluted to a ratio of 1:800 and Alexa Fluor 488 goat anti-rabbit (IgG, 111-545-003, Jackson Labs) diluted to a ratio of 1:800.

### 4.5. Time-Lapse Recording of Bone-Resorbing OCs

Time-lapse recordings of bone-resorbing OCs was performed using 0.2 thick bone slices (Boneslices.com) labeled with N-hydroxysuccinimide ester-activated rhodamine fluorescent dye (ThermoFisher Scientific). The matured OCs were reseeded onto these bone slices pre-incubated and labeled for 4 h in 96-well plates (1 × 10^5^ cells per well) with 100 nM SiR-actin (specifically binds to and labels f-actin) and 10 µM verapamil (Spirochrome, Cytoskeleton Inc., Denver, CO, USA) as described previously [15] or 100 nM SiR-700 lysosome tracker (fluorescently labeled peptide specifically binding cathepsin D) and 10 µM verapamil (Spirochrome, Cytoskeleton Inc.). Chloroquine was added to the relevant wells along with either SiR-actin or SiR-700 lysosome tracker. Subsequently, the labeled bone slices and OCs were placed inverted in a chambered cover glass well. The chambered cover glass was subsequently placed in a confocal microscope (FV10i (Olympus)) at 37 °C in 5% CO_2_ in a humidified atmosphere. OCs were imaged using a 10x objective and a confocal aperture of 2.0

## 5. Conclusions

The present study identifies two unique mechanisms involved in the trench resorption mode (Figure 8). (i) The ability to launch the trench resorption mode depends on whether the levels of active cathepsin K are sufficient, something which is highly variable amongst OCs generated from different individuals. Trench formation is therefore subjected to a great inter-individual variation (and is affected by age, gender, and menopause status). (ii) The ability to keep trench mode ongoing depends on a mechanism coordinating membrane/integrin recycling between the rear (exocytosis of resorption products) and the leading edge (progression of migration).

From a clinical perspective, it is interesting to note that the frequency of OCs making trenches seems to increase with the age of women, and that this increase is accompanied by elevated levels of active cathepsin K [21]. These observations make cathepsin K inhibitors very attractive anti-osteoporotic drugs, especially when considering the present findings. The ectosteric cathepsin K inhibitors [33,61] that are under development appear to be promising candidates since they do not show the side effects observed by active site inhibitors like odanacatib.

## Supplementary Materials

Supplementary materials can be found at https://www.mdpi.com/1422-0067/21/16/5924/s1.
